# Supplementary material for: BayesGmed: An R-package for Bayesian causal mediation analysis
Source: PLoS One. 2023 Jun 14;18(6):e0287037. doi: 10.1371/journal.pone.0287037 (PMC10266612; doi:10.1371/journal.pone.0287037)
Supplement: S1 Text — (DOCX) [file pone.0287037.s001.docx]

# S1 Supplementary text

## S 1. 1 Model formulation for the MUSICIAN study

Let $Y_{i}$represent the binary response variable (i.e., $Y_{i} = 1$ denoting a much or very much better outcome since randomization) of the ith subject, $A$ denotes the treatment ($A = 1$ for tCBT and $A = 0$ for TAU), $M_{ik}$ represent the kth mediator variable for the ith subject, and $\boldsymbol{Z}_{\boldsymbol{i}}$ denotes the baseline covariates to adjust for the ith subject. The outcome and mediator model of the mediation analysis is then formulated as follows

$\mathrm{logit}\left( P(Y_{i}=1|A_{i},M_{ik},\mathbf{Z}_{i}) \right)=\alpha_{0}+\boldsymbol{\alpha}_{Z}^{'}\mathbf{Z}_{i}+\alpha_{A}A_{i}+\alpha_{M}M_{ik},$

$E\left[ M_{ik} | \left( A_{i},\mathbf{Z}_{i} \right) \right]=\beta_{0}+\boldsymbol{\beta}_{Z}^{'}\mathbf{Z}_{i}+\beta_{A}A_{i}, with \epsilon_{i}\sim N(0, \sigma^{2}).$

The covariate vector $\boldsymbol{Z}_{i}$ includes age, gender, and baseline median GHQ score. We considered $K = 4$ mediators including $M1$ - tsk (fear of movement measure), $M2$ - active coping, $M3$ - passive coping, and $M4$ - sleep problems. We fit the above structural model for each mediators separately assuming the following priors.

$$\alpha= \left( \alpha_{0}, \alpha_{Z}, \alpha_{A}, \alpha_{M} \right)^{'}\sim MVN\left( location_{y}, scale_{y} \right),$$

$$\beta= \left( \beta_{0}, \beta_{Z}, \beta_{M} \right)^{'}\sim MVN(location_{m}, scale_{m}),$$

$$\sigma_{m}^{2}\sim(0, scale\_sd\_m)$$

We assume $\boldsymbol{0}_{6}$ and $\boldsymbol{0}_{5}$ for the $location_{y}$ and $location_{m}$, respectively. For the $scale_{y}$ and $scale_{m}$, we considered $10*\boldsymbol{I}_{6}$ and $10*\boldsymbol{I}_{5}$, respectively. Finally, we set the $scale\_sd\_m = 2.5$ for all models.

## S 1.2 R - code for the MUSICIAN study

Install and load the **BayesGmed**, **Rstan**, and **mediation** packages.

install.packages("rstan")

devtools::install_github("belayb/BayesGmed”)

install.packages("mediation")

library(rstan)

library(BayesGmed)

library(mediation)

**Mediation model fitting using the BayesGmed package with tsk (fear of movement) as a mediator.**

fit <- bayesgmed(outcome = "outcome",

mediator = "tsk",

treat = "TrT",

covariates = c("gender", "age","ghqmedian"),

dist.y = "binary", dist.m = "continuous",

link.y = "logit", link.m = "identity",

data = med_data,

priors = list(scale_m = 10*diag(5),

scale_y = 10*diag(6),

location_m = rep(0, 5),

location_y = rep(0, 6)),

iter=8000)

bayesgmed_summary(fit)

**Mediation model fitting using the mediation package with tsk (fear of movement) as a mediator.**

outcome_model <- glm(outcome ~ gender + age + ghqmedian +Trt + tsk+

data = med_data,

family = binomial(link="logit"))

mediator_model_tsk <- lm(tsk ~ gender + age + ghqmedian + Trt,

data = med_data)

med.out_tsk <- mediate(mediator_model_tsk, outcome_model_tsk,

treat = "Trt", mediator = "tsk",

robustSE = TRUE, sims = 100)

summary(med.out_tsk)

We repeat the above steps for each mediators.

**Sensitivity analysis for unmeasured confounding using the BayesGmed package with tsk (fear of movement) as a mediator.**

fit <- bayesgmed_sens(outcome = "outcome",

mediator = "tsk",

treat = "TrT",

covariates = c("gender", "age","ghqmedian"),

dist.y = "binary", dist.m = "continuous",

link.y = "logit", link.m = "identity",

data = med_data,

priors = list(scale_m = 10*diag(5),

scale_y = 10*diag(6),

location_m = rep(0, 5),

location_y = rep(0, 6),

location_gamma = rep(0,4),

scale_gamma = 0.5*diag(4)),

iter=8000)
