# Supplementary material for: BayesGmed: An R-package for Bayesian causal mediation analysis
Source: PLoS One. 2023 Jun 14;18(6):e0287037. doi: 10.1371/journal.pone.0287037 (PMC10266612; doi:10.1371/journal.pone.0287037)
Supplement: Attachment — Submitted filename: Response to Reviewers.docx [file pone.0287037.s002.docx]

Response to Comments from Reviewer 1

1. This paper focuses on an important research problem, i.e., mediation analysis. The goal of many studies in health is to understand how interventions produce changes in outcomes. While RCTs are useful for determining whether an intervention causes an outcome, they may not provide information on how the intervention works. Causal mediation analysis can help answer the "how" question by identifying the mechanisms by which interventions affect outcomes. The potential outcomes framework (POF) is commonly used for causal mediation analysis and has led to the development of different effect definitions, assumptions for estimating these effects, and methods for conducting sensitivity analyses. Two widely used approaches for estimating causal mediation effects are the regression-based approach and the simulation-based approach. While Bayesian mediation analysis has several advantages over frequentist methods, the software tools available have mainly focused on linear structural equation modelling (LSEM). This paper introduces a Bayesian estimation procedure and open-source software tool, BayesGmed, for causal mediation analysis using the Bayesian g-formula approach. The proposed method follows the POF for effect definition and identification and is illustrated using data from a randomized controlled trial.

   The paper presents an R-package for conducting causal mediation analysis, which can provide point and interval estimates for causal effects and sensitivity analyses around key assumptions. The proposed methodology was applied to data from a randomized controlled trial on the effects of tCBT on self-perceived change in health status in patients with chronic widespread pain. The results showed that tCBT had a significant effect on reducing sleep problems but none of the potential mediators was found to mediate the effect of tCBT. The paper suggests that improving the scope of tCBT or combining it with other interventions could increase patient benefit and that adequately powered trials are needed to explore these potential mediators and non-specific effects of tCBT.

   However, there are some limitations to the package, including the assumption of a parametric specification for the outcome and mediator model, and the consideration of only a single mediator without exposure mediator interaction. The authors plan to extend the package to handle these limitations in a future version, and users can also update the package for their own needs as it is distributed as open-source software.

   In summary, this paper is well-organised and solid in the technical part. I would accept it after a minor revision.

I would suggest authors add a section to introduce the related work in mediation analysis since enumerating related work helps to better declare the contribution of this paper.

**Response:** Thank you for the valuable feedback. Following the suggestion, we have added the following text in Paragraph 3 of the introduction section, highlighting the recent R-package development for mediation analysis within the Bayesian framework.

However, the open-source software tools developed so far, such as **bmlm** [Ref] and **bayestestR** [Ref], have mainly focused on the Bayesian implementation of the product-method or linear structural equation modelling (LSEM) approach [Ref]. The LSEM framework has been criticised for its limited applicability beyond specific statistical models. Recently, Rex et al., 2023 [Ref] introduced an R-package **bama**, which performs Bayesian mediation analysis based on the potential outcome framework. However, the **bama** only handles continuous exposure and outcome.

2. I list some references for your consider.
   1. "Causal mediation analysis with hidden confounders."
   2. "Disentangled Representation for Causal Mediation Analysis."

**Response:** Thank you for your suggestion. We have considered the suggestion citation and included in the updated manuscript.

Response to Comments from Reviewer 2

- Strengths:

  1. The authors address a significant gap in causal mediation analysis by proposing a Bayesian approach that overcomes the limitations of frequentist methods, particularly in small sample sizes.
  2. The introduction of the BayesGmed R-package is a valuable contribution to the field, providing an open-source tool for researchers and practitioners to use Bayesian causal mediation models.

  3. The application of the proposed methodology to the MUSICIAN study data effectively demonstrates the utility of the approach and provides a practical example for readers.

  4. The use of informative priors in the probabilistic sensitivity analysis is a useful addition, allowing researchers to assess the robustness of causal identification assumptions.

  The comparison of results obtained with BayesGmed and the mediation R-package strengthens the validity of the approach and highlights its potential advantages.

**Response:** Thank you for the insightful feedback.

- Weaknesses:
  1. The paper could benefit from a more detailed discussion of the advantages and disadvantages of Bayesian approaches compared to frequentist methods, providing readers with a better understanding of the implications of their choice.

**Response:** Thank you for the comment. Following the suggestion, we have added the following text in Paragraph 3 of the introduction section highlighting the advantage of Bayesian methods over the frequentist approach.

Compared to conventional frequentist mediation analysis, the Bayesian approach offers several advantages. First, Bayesian methods perform better when the sample size is small to moderate [ref], which is particularly common in clinical trials. Second, it enables the straightforward construction of credible intervals for direct and indirect effects, providing a probabilistic interpretation of the uncertainty surrounding the estimated effects. Third, it offers the option of conducting a probabilistic sensitivity analysis where bias parameters that reflect the investigators' beliefs about unmeasured confounders are incorporated as prior information [ref].

2. The authors mention that the mediated effects in the MUSICIAN study analysis were not statistically significant using BayesGmed. A more in-depth exploration of why this was the case and how this finding relates to the strengths of the Bayesian approach would be helpful.

**Response:** Thank you for the comment. We have suggested the difference observed here might be due to the superiority of Bayesian approach in the case of small sample size and small mediated effect as shown by [ref] through a simulation study. However, a similar simulation study is warranted to better understand the difference. To highlight this, we have added the following text in the discussion section.

The results obtained with both methods led to similar indirect effect estimates, except for the mediating effect of sleep problems, where the effect was found to be significant in the mediation R-package but not in BayesGmed. This discrepancy may be attributed to the small observed mediated effect in our study. As demonstrated by Yuan and MacKinnon (2009) in a simulation study, the Bayesian approach exhibits better 95% coverage than the frequentist approach when the sample size and the mediated effect is small. However, we recognize the need for a comprehensive simulation study to delve deeper into the comparison between BayesGmed and the mediation R-package

3. The paper could provide more guidance on selecting informative priors for the sensitivity analysis and discuss the potential pitfalls and biases introduced by using different priors.

**Response:** Thank you for the comment. We have added the following statement in the methods section.

The elicitation of δ (bias parameter), can be based on the investigators prior belief about the magnitude and direction of unmeasured confounding. In the absence of prior information on the direction or magnitude of unmeasured confounder, one can let the bias parameter to vary between zero and one, enabling an evaluation of continuous departures from the assumption of no unmeasured confounding assumption. For detailed discussion on the choice of informative priors for the sensitivity analysis we refer to McCandless et al., 2019[Ref].

Overall, the paper presents a valuable contribution to the field of causal mediation analysis by proposing a Bayesian approach and introducing an open-source software package for its implementation. While there are areas for improvement, the paper's strengths outweigh its weaknesses and provide a solid foundation for future research and practical applications.
